# Chronic Oxidative Stress, Mitochondrial Dysfunction, Nrf2 Activation and Inflammation in the Hippocampus Accompany Heightened Systemic Inflammation and Oxidative Stress in an Animal Model of Gulf War Illness

**DOI:** 10.3389/fnmol.2017.00182

**Published:** 2017-06-14

**Authors:** Geetha A. Shetty, Bharathi Hattiangady, Dinesh Upadhya, Adrian Bates, Sahithi Attaluri, Bing Shuai, Maheedhar Kodali, Ashok K. Shetty

**Affiliations:** ^1^Research Service, Olin E. Teague Veterans’ Medical Center, Central Texas Veterans Health Care System, TempleTX, United States; ^2^Institute for Regenerative Medicine, Texas A&M Health Science Center College of Medicine, Temple and College StationTX, United States; ^3^Department of Molecular and Cellular Medicine, Texas A&M Health Science Center College of Medicine, College StationTX, United States

**Keywords:** mitochondrial dysfunction, memory and mood impairment, mitogen-activated protein kinase, nuclear factor kappa b, NF-E2-related factor 2, oxidative stress, pro-inflammatory cytokines, reactive oxygen species

## Abstract

Memory and mood dysfunction are the key symptoms of Gulf war illness (GWI), a lingering multi-symptom ailment afflicting >200,000 veterans who served in the Persian Gulf War-1. Research probing the source of the disease has demonstrated that concomitant exposures to anti-nerve gas agent pyridostigmine bromide (PB), pesticides, and war-related stress are among the chief causes of GWI. Indeed, exposures to GWI-related chemicals (GWIR-Cs) and mild stress in animal models cause memory and mood impairments alongside reduced neurogenesis and chronic low-level inflammation in the hippocampus. In the current study, we examined whether exposure to GWIR-Cs and stress causes chronic changes in the expression of genes related to increased oxidative stress, mitochondrial dysfunction, and inflammation in the hippocampus. We also investigated whether GWI is linked with chronically increased activation of Nrf2 (a master regulator of antioxidant response) in the hippocampus, and inflammation and enhanced oxidative stress at the systemic level. Adult male rats were exposed daily to low-doses of PB and pesticides (DEET and permethrin), in combination with 5 min of restraint stress for 4 weeks. Analysis of the hippocampus performed 6 months after the exposure revealed increased expression of many genes related to oxidative stress response and/or antioxidant activity (*Hmox1, Sepp1*, and *Srxn1*), reactive oxygen species metabolism (*Fmo2, Sod2*, and *Ucp2*) and oxygen transport (*Ift172* and *Slc38a1*). Furthermore, multiple genes relevant to mitochondrial respiration (*Atp6a1, Cox6a1, Cox7a2L, Ndufs7, Ndufv1, Lhpp, Slc25a10*, and *Ucp1*) and neuroinflammation (*Nfkb1, Bcl6, Csf2, IL6, Mapk1, Mapk3, Ngf, N-pac*, and *Prkaca*) were up-regulated, alongside 73–88% reduction in the expression of anti-inflammatory genes *IL4* and *IL10*, and nuclear translocation and increased expression of Nrf2 protein. These hippocampal changes were associated with elevated levels of pro-inflammatory cytokines and chemokines (Tnfa, IL1b, IL1a, Tgfb, and Fgf2) and lipid peroxidation byproduct malondialdehyde in the serum, suggesting the presence of an incessant systemic inflammation and elevated oxidative stress. These results imply that chronic oxidative stress, inflammation, and mitochondrial dysfunction in the hippocampus, and heightened systemic inflammation and oxidative stress likely underlie the persistent memory and mood dysfunction observed in GWI.

## Introduction

Central nervous system (CNS) impairments, characterized by memory and mood dysfunction, are among the persistent symptoms in Gulf war illness (GWI) (Haley et al., 2000; Odegard et al., 2013; Rayhan et al., 2013a,b; Cooper et al., 2016). GWI is an enduring multi-symptom ailment, which currently affects an estimated 25–32% of the 700,000 veterans who served in Operation Desert Storm/Desert Shield in 1991 (Golomb, 2008; Institute of Medicine [IOM], 2010, 2014; White et al., 2016). The troops experienced exposure to a variety of chemicals during deployment, which included the intake of prophylactic drugs for variable durations for protection against nerve gas and other hazardous agents, widespread spraying and usage of mosquito repellants and pesticides to keep insects and rodents at bay in the desert region, exposure to low-level chemical warfare agents due to demolition of Iraqi facilities storing those agents, and smoke from oil-well fires (White et al., 2016). Thus, the concomitant exposure to pyridostigmine bromide (PB), which is an anti-nerve gas agent, and pesticides, such as N,N-diethyl-meta-toluamide (DEET) permethrin (PM), along with war-related stress or low-level exposures to chemical warfare agents, have been proposed as the chief causes of GWI (Haley and Kurt, 1997; Institute of Medicine [IOM], 2010, 2014; Steele et al., 2012; White et al., 2016).

The multiple chemical exposure theory has been supported by various animal studies. For example, we previously demonstrated that rats exposed to low-doses of GWI-related chemicals (GWIR-Cs), such as PB, DEET, and PM, and mild stress for 4 weeks consistently developed memory and mood impairments, which were akin to those observed in patients with GWI (Parihar et al., 2013; Hattiangady et al., 2014). Furthermore, these impairments developed in parallel to several pathological alterations in the hippocampus, which included the following: (i) persistently impaired neural stem cell proliferation in the neurogenic subgranular zone of the hippocampus; (ii) considerably reduced neurogenesis, i.e., the addition of new neurons, to the hippocampal circuitry underlying the formation of new memories and maintenance of normal mood function (Deng et al., 2010; Eisch and Petrik, 2012; Cameron and Glover, 2015); (iii) morphological signs of chronic low-level inflammation, typified by hypertrophy of astrocytes and activation of microglia (Parihar et al., 2013); (iv) loss of some glutamatergic neurons in several principal layers of the hippocampus (Parihar et al., 2013); and (v) reduction of hippocampal gamma-aminobutyric acid-synthesizing inhibitory interneurons (Megahed et al., 2015). Similar changes were also observed in other animal models of GWI, which utilized exposure to various doses and/or combinations of distinct GWIR-Cs (Abdullah et al., 2011, 2012; Ojo et al., 2014; O’Callaghan et al., 2015; Zakirova et al., 2015, 2016; Phillips and Deshpande, 2016).

Nonetheless, it is unknown whether the various morphological alterations observed in the hippocampus at extended time-points after the exposure to GWIR-Cs and stress are accompanied by molecular changes, such as alterations in genes encoding proteins that are relevant to increased oxidative stress, mitochondrial dysfunction, and enhanced inflammation. Therefore, in the current study, using quantitative real-time polymerase chain reaction (qRT-PCR) arrays, we first investigated whether exposure to GWIR-Cs and stress caused lasting modifications in the expression of genes related to oxidative stress response, metabolism of reactive oxygen species (ROS), oxygen transport, antioxidant activity, and inflammatory proteins in the hippocampus. Analyses was performed at a delayed time-point (6 months) after exposure to GWIR-Cs and stress in this study to simulate the scenario in GWI patients in whom over 25 years have passed since the exposure during PGW-1. A delay of 6 months after exposure in a rat model is equivalent to ∼17 years survival period after exposure in humans (Sengupta, 2013). Next, since mitochondrial dysfunction has been observed in the calf muscle of patients with GWI (Koslik et al., 2014), and alterations in mitochondrial respiration can be both the cause and the consequence of increased oxidative stress and inflammatory processes in neurodegenerative diseases (Friedland-Leuner et al., 2014; Currais, 2015), we examined potential alterations in the expression of genes related to mitochondrial respiration in the hippocampus. Since the expression of multiple genes relevant to oxidative stress and mitochondrial respiration was significantly upregulated, we investigated whether these changes were associated with activation of nuclear factor [erythroid-derived 2]-like 2 (Nrf2) in the hippocampus. Nrf2 is a transcription factor that is well recognized for its role in regulating antioxidant proteins that guard against oxidative damage elicited by injury or inflammation (Gold et al., 2012; Flowers et al., 2015). We confirmed Nrf2 activation by examining its nuclear translocation in hippocampal neurons and the concentration of activated Nrf2 in hippocampal lysates.

Finally, to determine whether the chronic increase in oxidative stress and inflammation in the hippocampus occurred in parallel to systemic inflammation and oxidative stress, we evaluated multiple pro-inflammatory proteins and malondialdehyde (MDA) in the serum. This analysis is particularly important because GWI patients exhibit increased concentration of multiple inflammatory biomarkers such as C-reactive protein, leptin, brain-derived neurotrophic factor and matrix metalloproteinase-9 in the blood (Johnson et al., 2016) and multiple other immune system irregularities (Skowera et al., 2004; Whistler et al., 2009; Broderick et al., 2013; Smylie et al., 2013; Khaiboullina et al., 2015). Additionally, a recent study has shown that multiple phospholipid species involved in inflammatory processes are elevated in the blood from GWI patients as well as rat and mouse models of GWI (Emmerich et al., 2017).

## Materials and Methods

### Animals

Young male Sprague-Dawley rats, aged 3–4 months, were obtained from Harlan Sprague-Dawley Inc. (Indianapolis, IN, United States) for this study. A combined institutional animal care and use committee of the Central Texas Veterans Health Care System and the Texas A&M Health Science Center College of Medicine approved all the experiments performed in the current study. Upon arrival, animals were housed with *ad libitum* access to commercial rat chow and water. Following 12–15 days of acclimatization in the vivarium, animals were randomly assigned to the naïve control, vehicle (VEH) control, or GWI groups, which here onward will be referred to as “naïve controls,” “VEH-rats,” and “GWI-rats,” respectively. GWI-rats received daily exposure to GWIR-Cs, namely, PB, DEET, and PM, and mild stress (5 min of restraint stress) for 4 weeks. In contrast, VEH-rats received sterile water (the vehicle solution used to dissolve PB), and 70% alcohol (the vehicle solution used to dissolve PB).

### Application of Chemicals and Restraint Stress

The dosages of PB, DEET, and PM, and duration of stress were based on prior studies of rat models of GWI (Abdel-Rahman et al., 2002, 2004; Parihar et al., 2013; Hattiangady et al., 2014; Megahed et al., 2015). PB (Sigma, St. Louis, MO, United States) was administered via oral gavage, at a dose of 1.3 mg/kg in 500 μl of sterile water. DEET and PM (Chem. Service Inc., West Chester, PA, United States) were dissolved in 70% alcohol, at dosages of 40 and 0.13 mg/kg, respectively, and applied dermally on the back of the animal’s neck. To facilitate direct application on the skin, the fur on the back of the neck and the upper thoracic region were shaved off, the exposed skin was wiped with 70% alcohol, and 200 μl each of the DEET and PM solutions were sequentially spread over the shaved skin using a short plastic pipette. Each animal was subjected to 5 min of restraint stress in a rat restrainer (Stoelting Research Instruments, Wood Dale, IL, United States), as detailed in our previous study (Parihar et al., 2013). Because DEET and PM are toxic drugs, these chemicals were handled and disposed as chemical hazards. Research personnel preparing these chemical solutions avoided skin contact, ingestion and inhalation by preparing them in a fume hood and using personal protective equipment (PPE) such as lab coat, safety goggles, and chemical resistant gloves. The personnel also used PPE while treating animals inside a fume hood.

### Collection of Hippocampal Samples for Quantitative Real-Time Polymerase Chain Reaction Studies

Each animal (*n* = 8/group) was deeply anesthetized with an overdose of isoflurane and decapitated, 6 months after the exposure to the GWIR-Cs and restraint stress. Following this, the brain was rapidly removed from the skull, snap frozen in dry ice, and stored at -80°C until further analysis. Following quick thawing, the entire hippocampus from both hemispheres of each brain was micro-dissected and processed for molecular biology studies, which comprised analyses of the expression of genes encoding proteins that are relevant for regulating oxidative stress, antioxidant activity, inflammation, and mitochondrial respiration, using qRT-PCR arrays.

### Extraction of Total RNA and Preparation of cDNA

Total RNA was first extracted from hippocampal samples belonging to GWI-rats and VEH-rats using RNeasy kit (Qiagen, Valencia, CA, United States; Cat#: 74104). We employed the manufacturer’s instructions, as previously detailed (Shetty et al., 2013). The concentration and quality of the RNA were determined by a Nanodrop spectrophotometer, using A260 and A260:A280 values, respectively (ThermoFisher Scientific, Wilmington, DE, United States). Total RNA (1 μg) was subsequently transcribed to cDNA using the RT2 First Strand Kit (Qiagen, Valencia, CA, United States; Cat# 330404) as previously detailed (Shetty et al., 2013). Briefly, 10 μl of genomic DNA elimination mixture was prepared using total RNA (2 μg for oxidative stress and 1 μg for inflammatory markers), 2 μl of genomic DNA elimination buffer, and nuclease free water. This mixture was incubated for 5 min at 42°C and chilled on ice. Following this, 10 μl of genomic DNA elimination mixture was gently mixed with 10 μl of RT cocktail, which contained 4 μl of BC3 (5X RT buffer 3), 1 μl of P2 (primer and external control mix), 2 μl of RE3 (RT enzyme mix 3), and nuclease free water. The cDNA synthesis reaction mixture was then incubated at 42°C for 15 min, before the reaction was stopped immediately by heating to 95°C for 5 min. Following the above reaction, 20 μl template cDNA was diluted with 91 μl of dH_2_O and stored at -20°C until further analysis.

### Measurement of Genes Related to Oxidative Stress and Mitochondrial Energy Metabolism

The Rat Oxidative Stress PCR Array used in this study (SABiosciences, Qiagen, Valencia, CA, United States; Cat#: PARN065Z) detected the expression of 84 key genes related to oxidative stress response, ROS metabolism, oxygen transport, and antioxidant activity in the hippocampus. A previously described (Shetty et al., 2013) qRT-PCR protocol was used. In brief, template cDNA (102 μl) from each hippocampal sample was mixed with 1,350 μl of 2X RT^2^ qPCR master mix (SABiosciences, Qiagen, Valencia, CA, United States) and 1,248 μl of dH_2_O to obtain a cocktail of 2,700 μl. This solution was then transferred to wells in a rat oxidative stress RT^2^ Profiler^TM^ PCR array plate (25 μl/well), which were comprised of pre-dispensed gene specific primer sets. In addition to primers for the 84 genes related to oxidative stress, each array also included primers for detecting five housekeeping genes, namely, beta-actin, beta-2-microglubulin, hypoxanthine phosphoribosyl transferase 1, lactate dehydrogenase A, and ribosomal protein lateral stalk subunit P1, and three RNA and three PCR quality controls. The reactions were performed according to the manufacturer’s protocol using a CFX96 Real-Time system (Bio-Rad, Hercules, CA, United States). The PCR amplification was done using the following two-step cycling program: 10 min denaturizing at 95°C, 40 cycles of 95°C for 15 s, and 60°C for 1 min. At the end of the amplification assay, a melt curve analysis was conducted to evaluate the specificity of the reaction. The conditions for the melt curve were 95°C for 1 min, followed by incremental increases of 0.5°C per 5 min, from 65 to 95°C.

The Rat Mitochondrial Energy Metabolism PCR Array (SABiosciences, Qiagen, Valencia, CA, United States; Cat#PARN008Z) used in this study profiled the expression of 84 key genes in the hippocampus, which encoded proteins that are relevant for mitochondrial respiration, such as genes encoding components of the electron transport chain and oxidative phosphorylation complexes. The reactions were performed as described above. For data analyses, the Ct (threshold cycle) values of all wells were exported to an Excel spreadsheet and analyzed using web-based SABiosciences PCR array data analysis software. The 2^ΔCt^ values for each gene were compared between VEH-rats and GWI-rats (*n* = 4–5/group).

### Measurement of Genes Related to Inflammation, Neurodegeneration, and Cognitive and Memory Function

The template cDNA (1 μl) from each hippocampal sample was mixed with 12.5 μl of 2X RT^2^ qPCR master mix, 1 μl of RT^2^ primer (10 μM stock, SABiosciences, Qiagen, Valencia, CA, United States), and 10.5 μl of dH_2_O to obtain a total volume of 25 μl. Primers relevant to selected genes (*n* = 35) encoding proteins involved in neurogenesis, learning and memory, and inflammation were used. Genes related to inflammation comprised the following: B-cell lymphoma 6 (*Bcl6*), complement component 3a receptor 1, chemokine (C-C-motif) ligand 5 (*Ccl5*, also known as *Rantes*), *Ccl17*, *Ccl22*, colony stimulating factor 2 (*Csf2*), interferon gamma (*Ifng*), insulin-like growth factor-binding protein 1 (*Igf1*), interleukin 1 alpha (*IL1a*), *IL1b, IL2, IL4, IL6, IL10, IL12b*, nuclear factor kappa B subunit 1 (*Nfkb1*), and tumor necrosis factor alpha (*Tnfa*). Genes that are relevant to neurodegeneration and/or cognitive and memory function included the following: mitogen-activated protein kinase 1 (*Mapk1*), *Mapk3*, protein kinase C alpha (*Prkaca*), glyoxylate reductase 1 homolog (*N-pac*), NMDA receptor regulated 2 (*Narg2*), nerve growth factor (*Ngf*), fibroblast growth factor-2 (*Fgfb*), vascular endothelial growth factor A (*Vegfa*), synaptotagmin-5 (*Syt5*), and forkhead box O3. Each array also contained two of three housekeeping genes glyceraldehyde-3-phosphate dehydrogenase, hypoxanthine phosphoribosyltransferase 1, and ribosomal protein lateral stalk subunit P1. The qRT-PCR reactions and data analyses were carried out as described above; 2^ΔCt^ values for each gene were compared between VEH-rats and GWI-rats(*n* = 6–8/group).

### Measurement of Nrf2 Using Nrf2-NeuN Dual Immunofluorescence and Confocal Microscopy

Six months after the exposure to GWIR-Cs and stress, GWI-rats and naïve controls (*n* = 8/group) were subjected to terminal anesthesia with isoflurane and intracardiac perfusion with 4% paraformaldehyde solution in phosphate buffer. Following this, the brain was carefully removed from the skull of each animal and post-fixed in 4% paraformaldehyde for approximately 16 h at 4°C. Next, brain tissues were treated with 30% sucrose solution in phosphate buffer until it sank to the bottom, and 30-μm-thick cryostat sections were cut coronally through the entire septo-temporal axis of the hippocampus. Sections were collected serially in 24-well plates filled with phosphate buffer. Every 20th section through the hippocampus was then processed from randomly chosen GWI-rats and naïve controls (*n* = 5/group) for Nrf2 and NeuN dual immunofluorescence. In brief, sections were first processed for Nrf2 immunofluorescence, which comprised sequential incubation of sections in 10% donkey serum for 30 min and rabbit anti-Nrf2 solution (1:500; Abcam, Cambridge, MA, United States) overnight. Following a wash in phosphate buffer, the sections were incubated in donkey anti-rabbit AF594 solution (1:200, ThermoFisher Scientific, Waltham, MA, United States) for 1 h and thoroughly washed with phosphate buffer thereafter. Next, sections were incubated in mouse anti-NeuN solution (1:1000; MilliporeSigma, Darmstadt, Germany) overnight, washed in phosphate buffer, and incubated in donkey anti-mouse AF488 solution (1:200; ThermoFisher Scientific) for 1 h. The sections were then thoroughly washed in phosphate buffer, and mounted on slides using SlowFade antifade mounting medium (ThermoFisher Scientific). Thereafter, 2-μm-thick Z-sectioning analysis was performed using confocal microscopy to visualize dense NeuN and speckled Nrf2 expression. Since Nrf2 expression was prominently seen in the CA3 subfield of the hippocampus in both GWI-rats and naïve controls, the percentage of CA3 pyramidal neurons displaying neuronal translocation of Nrf2 was quantified using multiple sections in each animal. In addition, since the number of Nrf2 speckles within nuclei varied in different groups, we also quantified the percentage of neurons expressing robust Nrf2 (having more than two speckles) among those expressing neuronal Nrf2. The values were compared between naïve control rats and GWI-rats (*n* = 5/group).

### Collection of Hippocampal and Serum Samples for Biochemical Studies

Six months after the exposure to GWIR-Cs and stress, additional GWI-rats and naïve controls (*n* = 8/group) were deeply anesthetized with an overdose of isoflurane. The heart was exposed and blood was quickly collected from the right atrium. Following this, the brain was rapidly removed from the skull, snap frozen in dry ice, and stored at -80°C. The serum was extracted from the collected blood using standard procedures and stored at -80°C. The entire hippocampi from both hemispheres were micro-dissected following quick thawing, and the samples were processed to measure Nrf2 levels through an ELISA. Serum samples were processed for measuring the relative concentration of various pro-inflammatory cytokines and chemokines, and MDA.

### Measurement of Hippocampal Nrf2 Concentration

Each hippocampal sample was weighed and lysed in 750 μl of ice-cold tissue extraction buffer (Life Technologies, Carlsbad, CA, United States) with protease inhibitors (Sigma–Aldrich Corp., St. Louis, MO, United States), using a sonic dismembrator for 10 s. The lysed solution was centrifuged twice (1,500 ×*g* at 4°C for 15 min) to avoid contamination with cellular debris. Aliquots of the supernatant solution were stored at -80°C until further use. The protein concentration of different samples was measured using a Pierce BCA reagent kit (ThermoFisher Scientific). We measured Nrf2 concentration in hippocampal lysates using the ELISA method, according to manufacturer instructions (Signosis, Santa Clara, CA, United States; Cat#: TE-0027). Briefly, hippocampal lysates were diluted to 10 μg/100 μl and added to each well of a 96-well plate that was pre-immobilized with the Nrf2 consensus sequencing oligonucleotide, which resulted in binding of activated Nrf2 in the whole cell lysate to the oligonucleotide. Samples were incubated with 50 μl of a specific antibody against an Nrf2 subunit for 1 h at room temperature (RT), washed, incubated with 50 μl of horseradish peroxidase-conjugated secondary antibody for 45 min at RT, washed again, and incubated with 50 μl of substrate solution for 10–30 min or until positive wells turned blue. Thereafter, 25 μl of stop solution was added, which transformed the color of the solution from blue to yellow. The yellow colored reaction product was measured using a Micro plate reader at 450 nm. The amount of Nrf2 in the lysate was directly proportional to the intensity of color. The values were compared between naïve control rats and GWI-rats (*n* = 6/group).

### Measurement of Cytokines and Chemokines in Serum

We used a rapid and sensitive rat cytokine array to measure16 cytokines/chemokines in the serum in a high-throughput manner, as per manufacturer instructions (Signosis; Cat#: EA4006). Briefly, serum samples (1:5 diluted) were dispensed to wells (100 μl per well) of 96-well plates that were coated with specific cytokine capture primary antibodies and incubated for 2 h at RT with gentle shaking. Following three washes with wash buffer, 100 μl of diluted biotin-labeled detection antibody mixture was added to each well and incubated for 1 h at RT with gentle shaking. Then, samples were washed and 100 μl of diluted streptavidin-HRP conjugate was added to each well and incubated for 45 min at RT. After three washes, 100 μl substrate was added to each well and incubated at RT. One hour later, 50 μl of stop solution was added to each well, which transformed the color of the solution from blue to yellow. The yellow reaction product was measured using a Micro plate reader at 450 nm. The concentration of the various cytokines and chemokines in the serum was directly proportional to the intensity of color in this procedure. The values for each cytokine or chemokine were compared between naïve control rats and GWI-rats (*n* = 6/group).

### Measurement of Malondialdehyde in the Serum

The level of MDA, which is a by-product of lipid peroxidation (a mechanism of cellular injury), in the serum was detected using an ELISA kit (LSBio, Seattle, WA, United States; Cat #: LS-F28018). Briefly, each well of the 96-well plate was pre-coated with an antigen-specific capture antibody. Diluted serum and known standards were added to each well followed by a fixed quantity of HRP-conjugated target antigen. The free antigen in the sample competed with the HRP conjugated antigen for binding to the capture antibody. Unbound antigen was washed away. A 3,3′,5,5′-tetramethylbenzidine substrate was then added which reacted with the HRP enzyme resulting in color development. Sulfuric acid stop solution was added to terminate the color development reaction. Then, the absorbance of the well was measured at a wavelength of 450 nm using a plate reader. A standard graph was plotted and the MDA concentration in unknown samples was computed from the best-fit curve. The values were expressed as ng/ml and compared between naïve control rats and GWI-rats (*n* = 6/group).

### Statistical Analysis

Prism software was used for all statistical analyses. Unpaired, two-tailed, Student’s *t*-test was employed for all comparisons between VEH and GWI or Naïve and GWI groups. When standard deviations were statistically significant between the two groups, a non-parametric test (Mann–Whitney test) was employed. Numerical data were presented as mean ± SEM and a *p*-value less than 0.05 was considered as statistically significant.

## Results

### Chronic Gulf War Illness Is Associated with Changes in the Expression of Genes That Respond to Oxidative Stress

**FIGURE 1:**
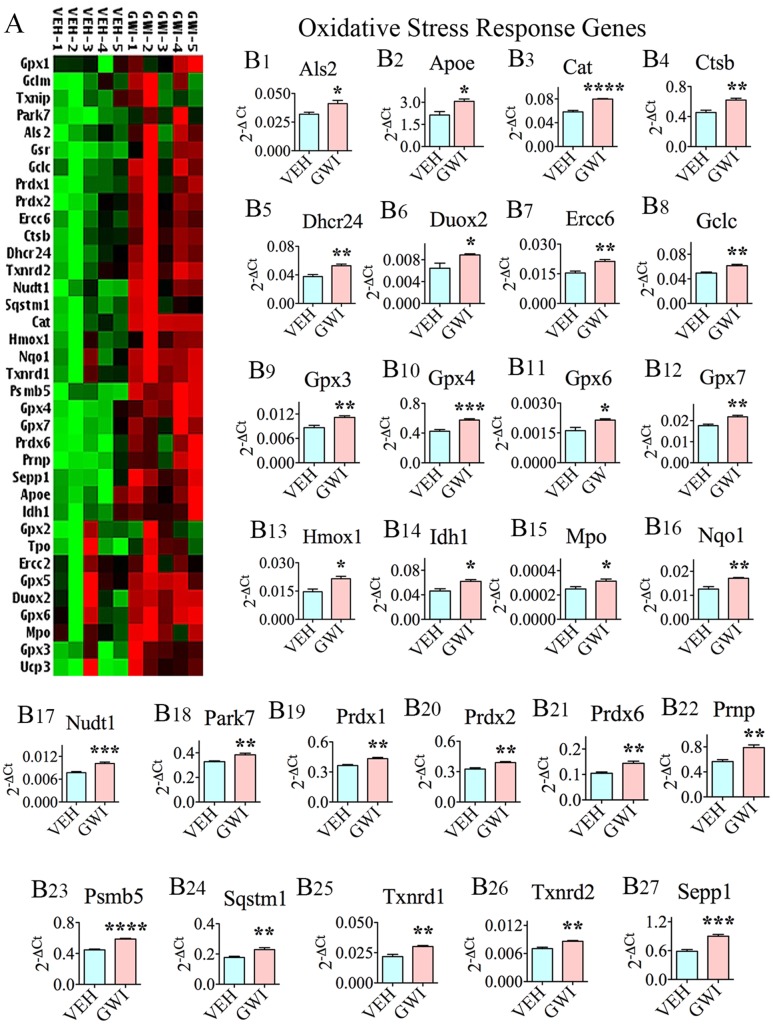
The hippocampus of rats with chronic gulf war illness-like symptoms (GWI-rats) presented an elevated expression of multiple oxidative stress-response genes. **(A)** Cluster diagram comparing the relative expression of genes that classically respond to enhanced oxidative stress, between animals exposed to vehicle (*n* = 5), GWI-related chemicals (GWIR-Cs), and stress (*n* = 5) when measured with 6 months after exposure using quantitative real-time polymerase chain reaction. Bar charts in **B1–B27** illustrate the elevated expression of 27 oxidative stress-response genes in animals exposed to GWIR-Cs and stress. ^∗^*p* < 0.05; ^∗∗^*p* < 0.01; ^∗∗∗^*p* < 0.001; ^∗∗∗∗^*p* < 0.0001.

The expression of a multitude of genes (*n* = 27) that typically respond to increased oxidative stress (referred to as oxidative stress-response genes) was significantly increased in the hippocampus, at 6 months after exposure to GWIR-Cs and mild stress (**Figures 1A,B1–B27**), which implied long-term changes in the expression of these genes. The genes were as follows: alsin rho guanine nucleotide exchange factor 2 (*Als2*; [B1]), apolipoprotein E (*ApoE*; [B2]), catalase (*Cat*; [B3]), cathepsin B (*Ctsb*; [B4]), 24-dehydrocholesterol reductase (*Dhcr24*; [B5]), dual oxidase 2 (*Duox2*; [B6]), excision repair cross-complementation group 6 (*Ercc6*; [B7]), glutamate-cysteine ligase catalytic subunit (*Gclc*; [B8]), glutathione peroxidase 3 (*Gpx3*; [B9]), *Gpx4* (B10), *Gpx6* (B11), *Gpx7* (B12), heme oxygenase 1 (*Hmox1*; [B13]), isocitrate dehydrogenase (*Idh1*; [B14]), myeloperoxidase (*Mpo*; [B15]), NAD(P)H quinone dehydrogenase 1 (*Nqo1*; [B16]), nudix hydrolase 1 (*Nudt1*; [B17]), parkinsonism associated deglycase (*Park7*; [B18]), peroxiredoxin1 (*Prdx1*; [B19]), *Prdx2* (B20), *Prdx6* (B21), prion protein (*Prnp*; [B22]), proteasome subunit beta 5 (*Psmb5*; [B23]), sequestosome 1 (*Sqstm1*; [B24]), thioredoxin reductase 1 (*Txnrd1*; [B25]), *Txnrd2* (B26), and Selenoprotein P Plasma 1 (*Sepp1*; [B27]).

### Chronic Gulf War Illness Is Allied with Increased Expression of Genes Involved in Reactive Oxygen Species Metabolism and Oxygen Transport

**FIGURE 2:**
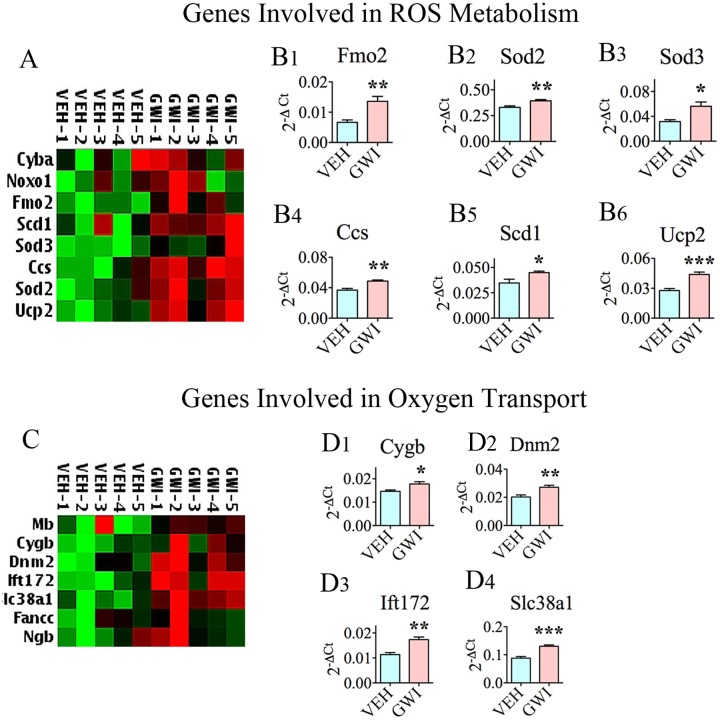
The hippocampus of rats with chronic gulf war illness-like symptoms (GWI-rats) exhibited an enhanced expression of genes encoding proteins involved in reactive oxygen species (ROS) metabolism and oxygen transport. **(A,C)** Are cluster diagrams comparing the relative expression of genes between animals exposed to vehicle (*n* = 5), GWI-related chemicals (GWIR-Cs), and stress (*n* = 5), which was measured 6 months after exposure using quantitative real-time polymerase chain reaction. Bar charts in **B1–B6** and **D1–D4** illustrate the elevated expression of genes involved in ROS metabolism and oxygen transport in animals exposed to GWIR-Cs and stress. ^∗^*p* < 0.05; ^∗∗^*p* < 0.01; ^∗∗∗^*p* < 0.001.

Six different genes encoding proteins that are involved in ROS metabolism were upregulated in the hippocampus at 6 months post-exposure to GWIR-Cs and mild stress (**Figures 2A,B1–B6**), suggesting the chronic presence of enhanced ROS in the hippocampus. This comprised the following genes: flavin containing monooxygenase 2 (*Fmo2*; [B1]), superoxide dismutase 2 (*Sod2*; [B2]), *Sod3* (B3), copper chaperone for superoxide dismutase (*Ccs*; [B4]), stearoyl-CoA desaturase 1 (*Scd1*; [B5]), and uncoupling protein 2 (*Ucp2*, [B6]). There were also increased expression of several genes involved in oxygen transport in the hippocampus at 6 months post-exposure to GWIR-Cs and stress (**Figures 2C,D1–D4**). These genes were the following: cytoglobin (*cygb*; [D1]), dynamin 2 (*Dnm2*; [D2]), intraflagellar transport 172 (*Ift172*, [D3]), and solute carrier family 38 member 1 (*Slc38a1*; [D4]).

### Chronic Gulf War Illness Is Linked with Enhanced Expression of Antioxidant Genes

**FIGURE 3:**
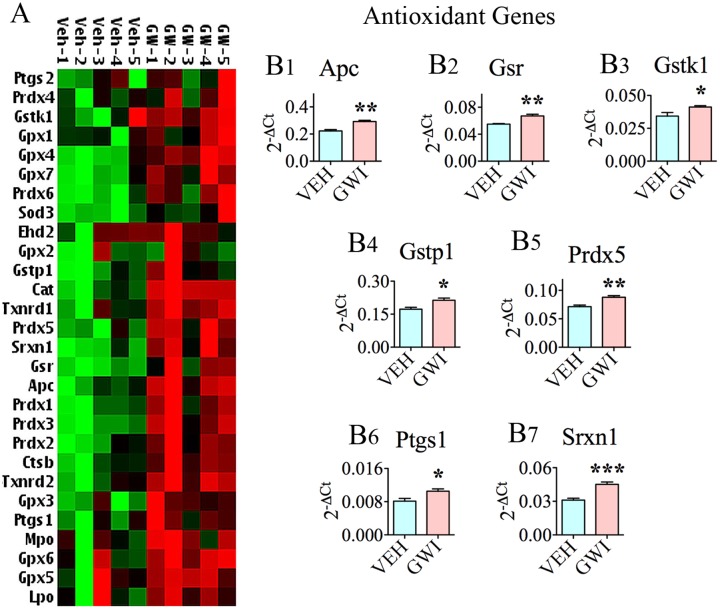
The hippocampus of rats with chronic gulf war illness-like symptoms (GWI-rats) showed a higher expression of multiple antioxidant genes. **(A)** Cluster diagram comparing the relative expression of antioxidant genes between animals exposed to vehicle (*n* = 5), GWI-related chemicals (GWIR-Cs), and stress (*n* = 5), which was measured 6 months after exposure using quantitative real-time polymerase chain reaction. Bar charts comparing the expression of *Apc*, *Gsr*, *Gstk1*, *Gstp1*, *Prdx5*, *Ptgs1*, and *Srxn1*, between the vehicle and GWI groups are illustrated in **B1–B7**. The bar charts comparing the expression of *Cat*, *Ctsb*, *Gpx3-4*, *Gpx6-7*, *Prdx1-2*, *Prdx6*, *Txnrd1*, and *Txnrd2*, between the vehicle and GWI groups are illustrated in **Figure 1**. ^∗^*p* < 0.05; ^∗∗^*p* < 0.01; ^∗∗∗^*p* < 0.001.

Many genes (*n* = 20) encoding proteins that are involved in antioxidant activity were upregulated in the hippocampus at 6 months post-exposure to GWIR chemicals and mild stress, further supporting the chronic presence of enhanced oxidative stress in the hippocampus (**Figure 3A**). The up-regulated expression of 12 of these genes are illustrated in **Figure 1**, which included *Cat* (B3), *Ctsb* (B4), *Gpx3, Gpx4*, *Gpx6*, and *Gpx7* (B9-B12), *Mpo* (B15), *Prdx1, Prdx2* and *Prdx6* (B19-B21), and *Txnrd1* and *Txnrd2* (B25-B26). The enhanced expression of the antioxidant gene *Sod3* is illustrated in **Figure 2B3**. Finally, there were seven additional genes with increased expression (shown in **Figure 3**), namely, adenomatous polyposis coli (*Apc*; [B1]), glutathione reductase (*Gsr*; [B2]), glutathione *S*-transferase kappa 1 (*Gstk1*, [B3]), glutathione *S*-transferase pi 1 (*Gstp1*; [B4]), *Prdx5* (B5), prostaglandin-endoperoxide synthase 1 (*Ptgs1*; [B6]), and sulfiredoxin 1 (*Srxn1*; [B7]).

### Chronic Gulf War Illness Exhibits Enhanced Expression of Genes Related to Mitochondrial Respiration

**FIGURE 4:**
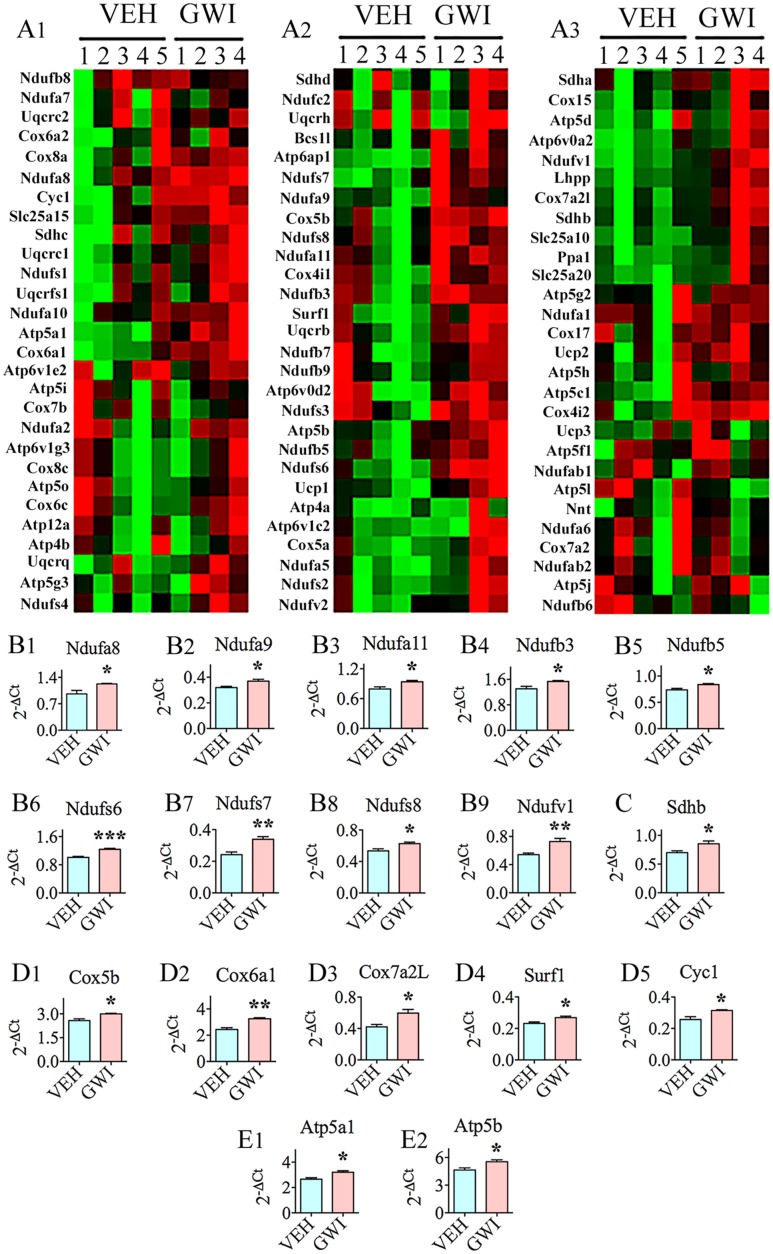
The hippocampus of rats with chronic gulf war illness-like symptoms (GWI-rats) presented an increased expression of multiple genes (*n* = 26) involved in mitochondrial respiration. **(A,C)** Are cluster diagrams comparing the relative expression of mitochondria-related genes between animals exposed to vehicle (*n* = 5), GWI-related chemicals (GWIR-Cs), and stress (*n* = 4), which was measured 6 months after exposure using quantitative real-time polymerase chain reaction. Bar charts in **B1–B9,C,D1–D5,E1,E2** illustrate the elevated expression of genes involved in mitochondrial complex I (*Ndufa8, Ndufa9, Ndufa11, Ndufb3, Ndufb5, Ndufs6, Ndufs7, Ndufs8*, and *Ndufv1*; **B1–B9**), complex II (*Sdhb*; **C**), complex IV (*Cox5b, Cox6a1, Cox7a2L, Surf1*, and *Cyc1*; **D1–D5**), and complex V (*Atp5a1 and Atp5b*; **E1,E2**) of GWI-rats. ^∗^*p* < 0.05; ^∗∗^*p* < 0.01; ^∗∗∗^*p* < 0.001.

Many genes (*n* = 26) involved in mitochondrial respiration were up-regulated in the hippocampus, 6 months post-exposure to GWIR-Cs and mild stress (**Figures 4A1–A3**). These comprised genes encoding proteins that are important for the function of complexes I, II, IV, and V of the mitochondrial respiratory chain (**Figures 4B1–B9,C,D1–D5,E1,E2**). The nine genes with altered expression pertaining to complex I were NADH dehydrogenase [ubiquinone] 1 alpha subcomplex 8 (*Ndufa8*, [B1]), *Ndufa9* [B2], *Ndufa11* [B3], NADH dehydrogenase [ubiquinone] 1 beta subcomplex 3 (*Ndufb3*, [B4]), *Ndufb5* [B5], NADH dehydrogenase [ubiquinone] Fe-S protein 6 (*Ndufs6*, [B6]), *Ndufs7* [B7], *Ndufs8* [B8], and NADH dehydrogenase [ubiquinone] flavoprotein 1 (*Ndufv1*, [B9]). The only gene that was modified in relation to complex II function was succinate dehydrogenase complex, subunit B, iron sulfur (*Sdhb*; [C]). The five genes associated with complex IV (cytochrome C oxidase – the terminal component of mitochondrial respiratory chain) were cytochrome C oxidase subunit Vb (*Cox5b*; [D1]), cytochrome C oxidase subunit V1a polypeptide 1 (*Cox6a1*; [D2]), cytochrome C oxidase subunit VIIa polypeptide 2 like (*Cox7a2L*; [D3]), surfeit 1 (*Surf1*; [D4]), and cytochrome C-1 (*Cyc1*; [D5]). The two genes relevant to complex V (mitochondrial ATP synthase, the enzyme that converts ADP to ATP), which had an altered expression, were ATP synthase, H+ transporting, mitochondrial F1 complex, alpha subunit 1 (*Atp5a1*; [E1]) and *Atp5b* (E2).

**FIGURE 5:**
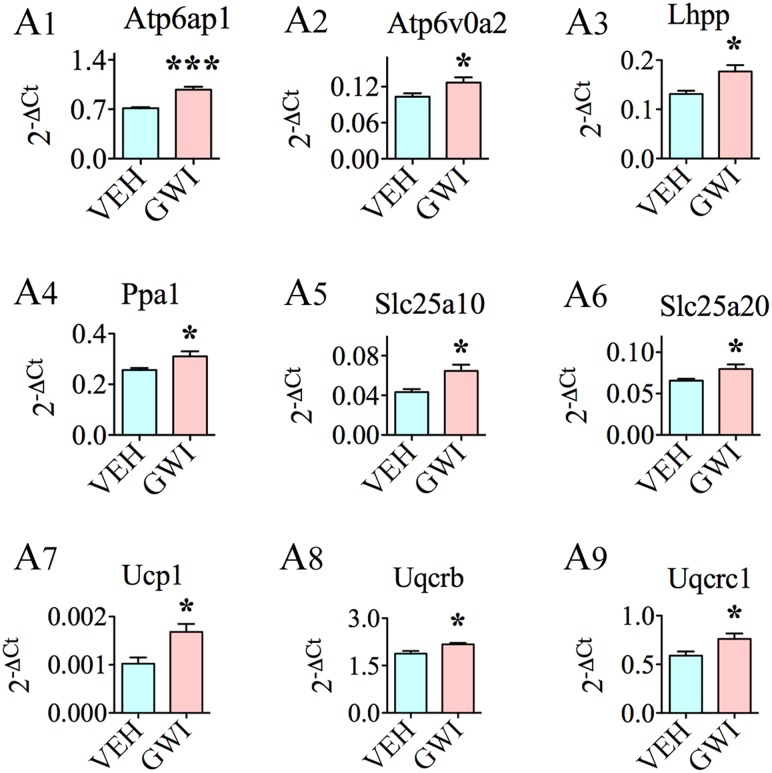
The hippocampus of rats with chronic gulf war illness-like symptoms (GWI-rats) showed an increased expression of nine other genes relevant to mitochondrial or other organelle function when measured 6 months after exposure to GWI-related chemicals and stress by quantitative real-time polymerase chain reaction. Bar charts **(A1–A9)** illustrate genes that showed elevated expression in animals exposed to GWIR-Cs and stress (*n* = 4–5/group). ^∗^*p* < 0.05; ^∗∗∗^*p* < 0.001.

In addition to the above genes, nine other genes relevant to mitochondrial/other organelle function displayed enhanced expression (**Figure 5**). These included the following: ATPase, H+ transporting, lysosomal accessory protein 1 (*Atp6ap1*; [A1]), ATPase, H+ transporting, lysosomal V0 subunit A2, involved in acidification of cell organelles (*Atp6voa2*; [A2]), phospholysine phosphohistidine inorganic pyrophosphate phosphatase, involved in respiratory electron transport (*Lhpp*; [A3]), pyrophosphatase [inorganic] 1, important for phosphate metabolism of cells (*Ppa1*; [A4]), solute carrier family 25 member 10, one of mitochondrial membrane carrier proteins (*Slc25a10*; [A5]), solute carrier family 25 member 20, another mitochondrial membrane carrier protein (*Slc25a20*; [A6]), uncoupling protein 1, one of mitochondrial anion carrier proteins (*Ucp1*; [A7]), ubiquinol-cytochrome C reductase binding protein, participates in the transfer of electrons when ubiquinone is bound (*Uqcrb*; [A8]), and ubiquinol-cytochrome C reductase core protein I, involved in metabolism (*Uqcrc1*; [A9]).

### Chronic Gulf War Illness Is Associated with Enhanced Expression of Genes Involved in Inflammation and Neurodegenerative Disorders

**FIGURE 6:**
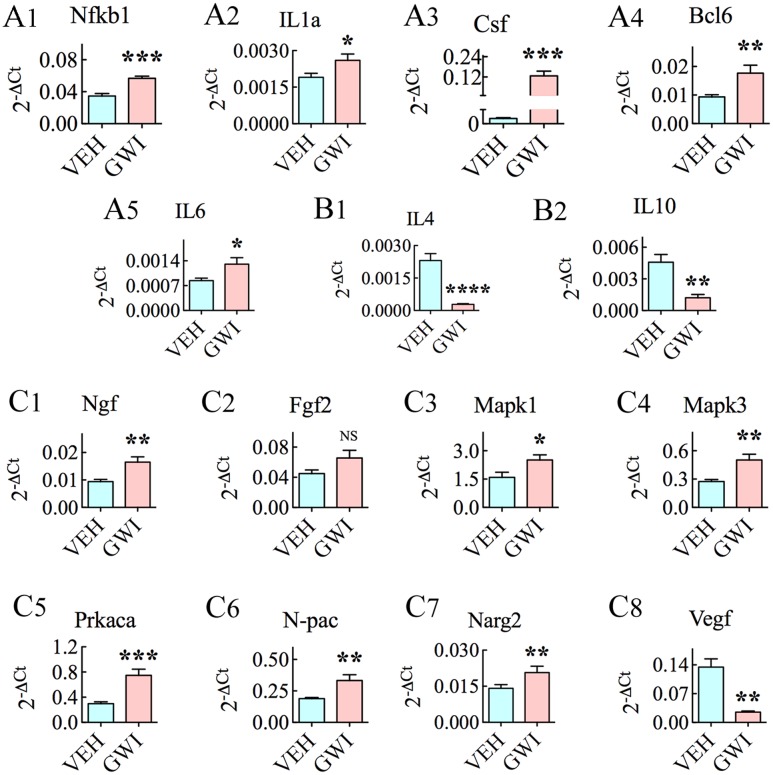
The hippocampus of rats with chronic gulf war illness-like symptoms (GWI-rats) displayed an increased expression of genes involved in inflammatory activity (*Nfkb1, IL1a, Csf, Bcl6, and IL6*, **A1–A5**), decreased expression of genes relevant to anti-inflammatory activity (*IL4 and IL10*, **B1,B2**), enhanced expression of genes associated with neurodegenerative disorders and/or cognitive and memory function (*Ngf, Fgfb, Mapk1, Mapk3, Prkaca, N-pac*, and *Narg2*, **C1–C7**) and decreased expression of a gene involved in angiogenesis (*Vegf*, **C8**), when measured 6 months after exposure to GWI-related chemicals and stress by quantitative real-time polymerase chain reaction (*n* = 6–8/group). ^∗^*p* < 0.05; ^∗∗^*p* < 0.01 ^∗∗∗^*p* < 0.001, ^∗∗∗∗^*p* < 0.0001.

Many genes linked to inflammation, namely, *Nfkb1*, *IL1a*, *Csf2*, *Bcl6*, and *IL6* were upregulated in the hippocampus at 6 months after exposure to GWIR-Cs and stress (**Figures 6A1–A5**). The increased expression of these pro-inflammatory genes was associated with the decreased expression of anti-inflammatory genes *IL4* and *IL10* (*p* < 0.01–0.0001, **Figures 6B1,B2**). Further, several genes associated with neurodegenerative disorders and/or cognitive and memory function also had an altered expression. These included the increased expression of *Ngf*, *Fgfb*, *Mapk1*, *Mapk3*, *Prkaca*, *N-pac*, and *Narg2* (**Figures 6C1–C7**), and the decreased expression of *Vegfa* (**Figure 6C8**).

### Chronic Gulf War Illness Is Linked with Nrf2 Activation in Hippocampal Neurons

**FIGURE 7:**
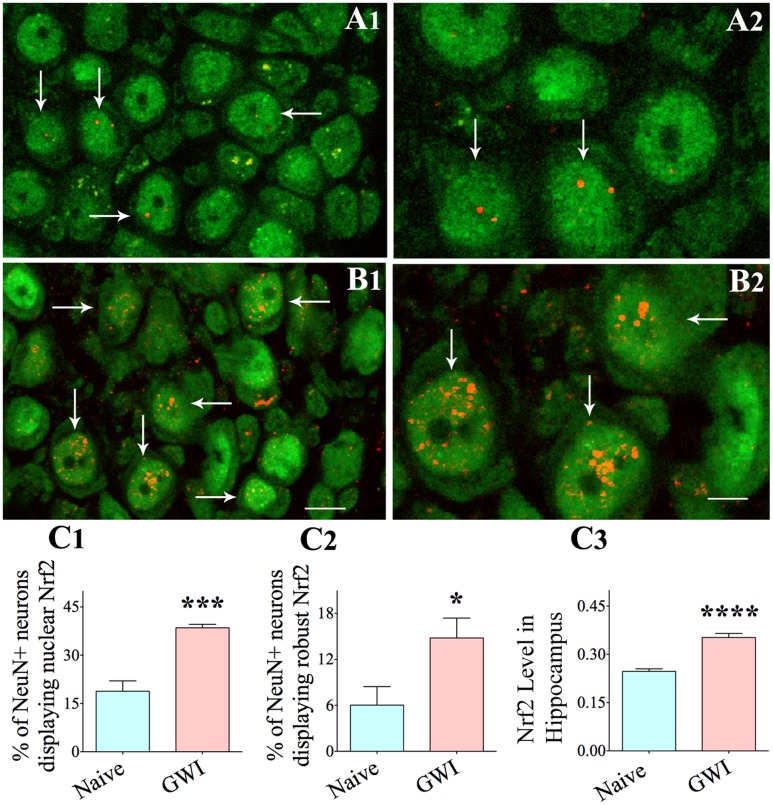
The hippocampus of rats with chronic gulf war illness-like symptoms (GWI-rats) exhibited an increased Nrf2 expression when evaluated 6 months after exposure to GWI-related chemicals and stress. Figures **(A1–A4)** illustrate examples of neuron specific nuclear antigen positive (NeuN+) hippocampal CA3 pyramidal neurons (green) displaying nuclear translocation of Nrf2 (red particles) in a naïve control animal (arrows in **A1,A2**) and in an animal exposed to GWI-related chemicals and stress (arrows in **B1,B2**). Note that nuclear translocation of Nrf2 is increased in the animal exposed to GWI-related chemicals and stress **(B1,B2)**. Bar charts in **C1–C3** compare percentages of NeuN+ hippocampal neurons displaying nuclear Nrf2 **(C1)**, percentages of hippocampal neurons showing robust nuclear Nrf2 **(C2)** and the extent of activated Nrf2 measured through ELISA in the entire hippocampus between age-matched naïve control animals and animals exposed to GWI-related chemicals and stress (*n* = 5–6/group). Scale bar, **(A1,B1)**, 10 μm; **(A2,B2)**, 5 μm. ^∗^*p* < 0.05 ^∗∗∗^*p* < 0.001, ^∗∗∗∗^*p* < 0.0001.

Using confocal microscopy and dual immunofluorescence, we examined if NeuN + neurons in the hippocampus expressed nuclear Nrf2 (activated Nrf2) (**Figure 7**). Since the frequency of neurons with nuclear Nrf2 was greatest in the CA3 hippocampal in GWI-rats, we chose this subfield for quantification. In naïve controls, neurons expressed nuclear Nrf2 infrequently. Further, neurons positive for Nrf2 displayed minimal Nrf2 expression (one or two speckles) in the nucleus (**Figures 7A1,A2,C1**). In comparison, more CA3 pyramidal neurons expressed nuclear Nrf2 in GWI-rats (**Figure 7B1**). The overall amount of nuclear Nrf2 in these neurons was also greater, which was typically evidenced by three or more speckles of positive staining (**Figure 7B2**). GWI-rats had significantly more neurons with nuclear Nrf2 (**Figure 7C1**, *p* < 0.001) and a greater fraction of neurons with robust nuclear Nrf2 (i.e., >three speckles of variable size, **Figure 7C2**, *p* < 0.05). To validate the activation of Nrf2 in the hippocampus, we performed an ELISA for activated Nrf2 using hippocampal tissue lysate. The overall amount of activated Nrf2 was greater in the hippocampus of GWI-rats, in comparison to naïve controls (*p* < 0.0001, **Figure 7C3**). Thus, we confirmed that chronic GWI was associated with considerable activation of Nrf2 in the hippocampus.

### Chronic Gulf War Illness Is Associated with Increased Concentration of Multiple Cytokines, Chemokines, and an Oxidative Stress Marker in the Serum

**FIGURE 8:**
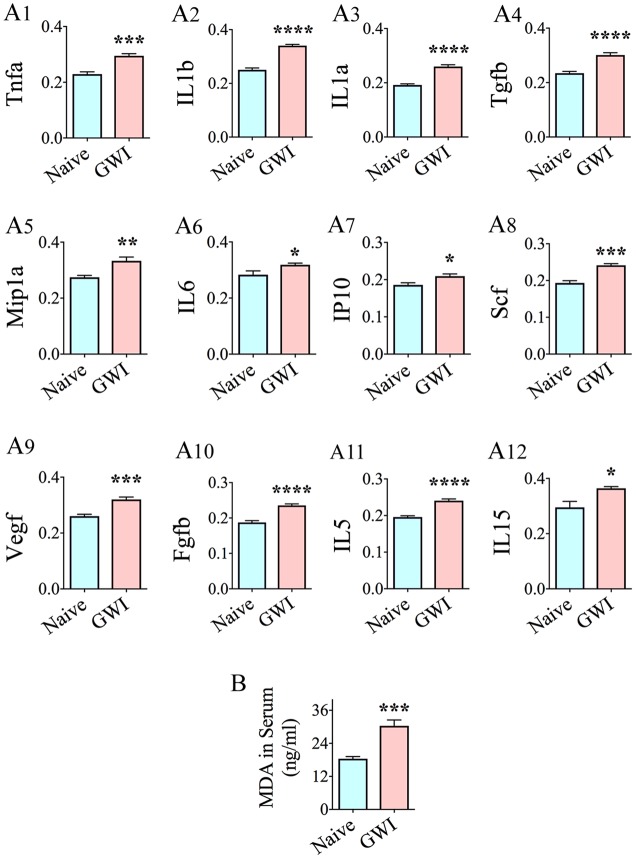
Animals with chronic gulf war illness-like symptoms (GWI-rats) showed increased levels of multiple pro-inflammatory cytokines and chemokines, and malondialdehyde (MDA) in serum when examined 6 months after exposure to GWI-related chemicals and stress. Bar charts **A1–A12** compare the relative levels of cytokines and chemokines between naïve control rats and GWI-rats (*n* = 6/group). Note the increased levels of Tnfa, IL1b, IL1a, Tgfb, MIP1a, IL6, IP10, Scf, Vegf, Fgfb, IL5, and IL15 **(A1–A12)** in GWI-rats, in comparison to age-matched naive control rats. Bar chart **B** illustrates an elevated concentration of MDA in the serum of GWI-rats, in comparison to naïve control rats (*n* = 6/group). ^∗^, *p* < 0.05; ^∗∗^*p* < 0.01; ^∗∗∗^*p* < 0.001; ^∗∗∗∗^*p* < 0.0001.

Many cytokines and chemokines were elevated in the serum, 6 months after exposure to GWIR-Cs and stress (**Figure 8**). These were the following: Tnfa, IL1b, IL1a, transforming growth factor beta (Tgfb), macrophage inflammatory protein 1 alpha (Mip1a, also called CCL3), IL6, interferon gamma-induced protein 10 (IP10, also called Cxcl10), stem cell factor, a cytokine that binds to c-KIT receptor (Scf), Vegf, Fgfb, IL5, and IL15 (**Figures 8A1–A12**). Cytokines/chemokines that were unchanged included macrophage chemoattractant protein 1 (Mcp1), Ccl5 (or Rantes), Ifng, and leptin. The concentration of MDA (a by-product of lipid peroxidation and increased oxidative stress) was also greatly enhanced in the serum of GWI-rats (**Figure 7B**). Thus, multiple cytokines and chemokines were upregulated, at a systemic level, in association with the increased oxidative stress in a rat model of chronic GWI.

## Discussion

This study provided first evidence that exposure to GWIR-Cs and stress leads to chronic oxidative stress, mitochondrial dysfunction, Nrf2 activation, and inflammation in the hippocampus, a region of the brain important for learning, memory, and mood but showing persistent impairment in animal models of GWI (Parihar et al., 2013; Hattiangady et al., 2014; Zakirova et al., 2015; Abdullah et al., 2016). Moreover, these adverse alterations in the hippocampus were not seen in isolation, but rather were associated with systemic inflammation and oxidative stress.

Multiple genes that classically respond to heightened oxidative stress in the environment displayed increased expression. These comprised 27 oxidative stress-response genes (*Als2, Apoe, Cat, Ctsb, Dhcr24, Duox2, Ercc6, Gclc, Gpx3-4, Gpx6-7, Hmox1, Idh1, Mpo, Nqo1, Nudt1, Park7, Prdx1-2, Prdx6, Prnp, Psmb5, Sqstm1, Txnrd1-2*, and *Sepp1*), 6 genes encoding proteins involved in ROS metabolism (*Fmo2, Sod2, Sod3, Ccs, Scd1*, and *Ucp2*), 4 genes coding proteins involved in oxygen transport (*Cygb, Dnm2, Ift172*, and *Slc38a1*), and 20 antioxidant genes (*Apc, Cat, Ctsb, Gpx3-4, Gpx6-7, Gsr, Gstk1, Gstp1, Mpo, Prdx1-2, Prdx5-6, Ptgs1, Txnrd1-2, Sod3*, and *Srxn1*). The proteins encoded by these genes serve multiple functions, some of which are briefly outlined in **Table 1**. Among genes that were statistically up-regulated (**Table 1**), 8 genes displayed the highest levels of expression (1.5-fold or greater increase). These include *Fmo2, Hmox1, Ift172, Sepp1, Slc38a1, Sod3, Srxn1*, and *Ucp2.* Greatly enhanced expression of these genes implied a compensatory reaction against oxidative stress that prevailed in the hippocampus of GWI-rats because, proteins coding some of these genes are directly involved in the regulation of ROS production (*Fmo2*, Mao et al., 2010), oxidative cleavage of pro-oxidant damaging molecule heme (*Hmox1*, Xu et al., 2016), transport of oxygen (*Ift172*, Gorivodsky et al., 2009), extracellular antioxidant activity (*Sepp1, Sod3*, Huang et al., 2015; Davis et al., 2017), modulation of intracellular ROS (*Slc38a1*, Ogura et al., 2011), oxidoreductase activity (*Srxn1*, Zhou et al., 2015), and uncoupling of oxidative phosphorylation to reduce mitochondrial ROS (*Ucp2*, Graw et al., 2015). Furthermore, some of the up-regulated genes also encode proteins associated with NF-kB signaling and Nrf2 pathway (*Psmb5* and *Sqstm1*), inflammation (*Mpo*), neuroprotection (*Cat, Cygb, Dhcr24*, and *Prnp*), neurocognitive disorders (*Apc* and *Ctsb*), Alzheimer’s disease (*Dhcr24, Dnm2*, and *Nqo1*), amyotrophic lateral sclerosis (*Als2*), hippocampal resting state functional connectivity (*Apoe*), reduced neurogenesis (*Mpo*), anti-apoptotic activity (*Dhcr24*), axon growth and vesicular trafficking (*Dnm2*), cellular detoxification and stress response (*Gstk1* and *Gstp1*), DNA repair (*Ercc6*), and altered presynaptic glutamatergic function (*Slc38a1*). From these, it appears that persistent oxidative stress and inflammation in the hippocampus caused multiple adverse effects on hippocampus function. Persistently reduced neurogenesis, and hippocampus-dependent cognitive and memory impairments are among the conspicuous symptoms observed in GWI-rats (Parihar et al., 2013; Hattiangady et al., 2014). However, the presence of altered glutamatergic neurotransmission and resting state functional connectivity are yet to be confirmed.

**Table 1:** Genes related to oxidative stress response.

Gene name	Encoded protein and function	Reference
Als2	Alsin rho guanine nucleotide exchange factor 2 activates Ras superfamily of GTPases and participates in amyotrophic lateral sclerosis pathway	Hadano et al., 2016
Apc	Adenomatous polyposis coli is involved with cell adhesion, regulation of synapse maturation and cognition	Mohn et al., 2014; Hickman et al., 2015
Apoe	Apolipoprotein E mediates plasma lipoprotein transport and modulates hippocampal resting state functional connectivity	Shen et al., 2017
Cat	Catalase protein is an antioxidant that converts hydrogen peroxide to water and oxygen and protects against neurotoxicity	Wang et al., 2003
Ccs	Copper chaperone for superoxide dismutase is a chaperone that inserts copper into the copper zinc Sod1. Exposure to neurotoxic chemicals elevates Ccs expression	Kim et al., 2011
Ctsb	Cathepsin B is involved in the proteolytic processing of amyloid precursor protein, autophagy and neurocognitive disorders	Siangphoe and Archer, 2015
Cygb	Cytoglobin catabolizes H_2_O_2_ and lipid hydroperoxides and its expression is increased in the brain following chemical exposure or injury as a neuroprotective mechanism	Tian et al., 2013
Dhcr24	24-Dehydrocholesterol reductase has delta 24 sterol reductase, oxidoreductase, anti-apoptotic and neuroprotective activity.	Peri et al., 2011; Le Duc et al., 2015
Dnm2	Dynamin 2 regulates neuron morphology, axon growth, and vesicular trafficking processes such as endocytosis. Reduced expression is linked with increased Abeta secretion in Alzheimer’s disease.	Kamagata et al., 2009
Duox2	Dual Oxidase 2 is an NADPH oxidase involved in the generation of ROS.	Konior et al., 2014
Ercc6	Excision Repair cross complement group 6 is a chromatin binding and DNA binding protein involved in DNA repair.	Baas et al., 2010
Fmo2	Flavin containing monooxygenase 2 regulates the production of ROS.	Mao et al., 2010
Gclc	Glutamate-cysteine ligase catalytic subunit is a rate limiting enzyme in the synthesis of antioxidant glutathione.	Casañas-Sanchez et al., 2016
Gpx3-4,6-7	Glutathione peroxidase proteins 3–4 and 6–7 are involved in detoxification of H_2_O_2_	Roseni Mundstock Dias et al., 2014
Gsr	Glutathione reductase converts glutathione and NADP+ to glutathione disulphide and NADPH.	Casañas-Sanchez et al., 2016
Gstk1	Glutathione *S*-transferase kappa 1 is involved in cellular detoxification and stress response.	Lo et al., 2008
Gstp1	Glutathione *S*-transferase pi 1 is involved in cellular detoxification and stress response.	Lo et al., 2008
Hmox1	Heme oxygenase mediates oxidative cleavage of heme.	Xu et al., 2016
Idh1	Isocitrate dehydrogenase 1 converts isocitrate to a-ketoglutarate with concomitant reduction of NADP(+) to NADPH.	Chen et al., 2016
Ift172	Intraflagellar Transport 172 is an oxygen transporter that mediates intraflagellar transport, a process necessary for the genesis and maintenance of cilia.	Gorivodsky et al., 2009
Mpo	Myeloperoxidase, an oxidizing enzyme secreted by activated leukocytes, is increased in neurodegenerative, increased oxidative stress or inflammatory conditions.	Kim et al., 2016
Nqo1	NAD(P)H Quinone Dehydrogenase 1 is a detoxifying enzyme that is elevated in the brain in neurotoxic conditions and early stage Alzheimer’s disease.	Torres-Lista et al., 2014
Nudt1	Nudix Hydrolase1 catalyzes the hydrolysis of oxidized purine nucleoside triphosphates, such as 8-oxo-dGTP, to monophosphates to prevent disincorporation in cellular DNA and RNA.	Kajitani et al., 2006
Park 7	Parkinsonism Associated Deglycase also called DJ-1 plays a protective role against oxidative stress and mutation of Park7 gene causes familial Parkinson’s disease.	Lev et al., 2013
Prdx1-2,6	Peroxiredoxin proteins reduce H_2_O_2_ and alkyl hydroperoxides and are elevated in conditions of amplified oxidative stress.	Richter et al., 2014
Prnp	Prion gene is involved in conformational conversion of prion and in neuroprotection.	Jeong et al., 2012
Psmb5	Proteasome Subunit Beta 5 is involved in the degradation of abnormal intracellular proteins and plays a protective role against oxidative damage through the Nrf2-antioxidant responsive element (ARE) pathway.	Kwak and Kensler, 2006
Ptgs1	Prostaglandin-endoperoxide synthase 1 is involved in the conversion of arachinodate to prostaglandin and functioning as a cyclooxygenase and a peroxidase.	Tang et al., 2012
Scd1	Stearoyl-CoA desaturase 1 participates in the synthesis and regulation of unsaturated fatty acids.	Cintra et al., 2012
Sepp1	Selenoprotein P is an extracellular antioxidant that transport selenium.	Huang et al., 2015
Slc38a1	Solute carrier family 38 member 1 is involved in Na(+)-dependent glutamine transport and modulates presynaptic glutamatergic function and intracellular ROS.	Ogura et al., 2011
Sod 2,3	Superoxide dismutase 2 is manganese Sod, located in mitochondrial spaces whereas Sod3 is an extracellular isoform, and both are potent antioxidant enzymes.	Lee et al., 2012; Davis et al., 2017
Sqstm1	Sequestosome 1is a multifunctional protein that binds to ubiquitin and regulates activation of the nuclear factor kappa-B (NF-kB) signaling pathway and Nrf2 antioxidant system.	Hashimoto et al., 2016
Srxn1	Sulfiredoxin 1 has oxidoreductase activity.	Zhou et al., 2015
Txnrd1–2	Thioredoxin Reductase 1–2 are cytosolic antioxidants that reduce thioredoxins and protect against oxidative stress.	Tohidnezhad et al., 2014
Ucp2	Uncoupling protein 2 is involved in uncoupling of oxidative phosphorylation to reduce ROS metabolism in mitochondria.	Graw et al., 2015

The presence of chronic oxidative stress in the hippocampus was also supported by changes in the expression of genes related to mitochondrial respiration. These genes particularly, encoded proteins associated with the function of complex I (*Ndufa8, Ndufa9, Ndufa11, Ndufb3, Ndufb5, Ndufs6, Ndufs7, Ndufs8*, and *Ndufv1*), complex II (*Sdhb*), complex IV (*Cox5b, Cox6a1, Cox7a2L, Surf1*, and *Cyc1*), and complex V (*Atp5a1* and *Atp5b*) of the mitochondrial respiratory chain. Furthermore, nine genes, namely, *Atp6ap1, Atp6voa2, Lhpp, Ppa1, Slc25a10, Slc25a20, Ucp1, Uqcrb*, and *Uqcrc1*, which are relevant to mitochondrial/other organelle function also showed elevated expression. Among genes that were statistically up-regulated, eight genes displayed higher levels of expression (1.3-fold or greater increase). These include *Atp6a1, Cox6a1, Cox7a2L, Ndufs7, Ndufv1, Slc25a10*, and *Ucp1*. Mitochondrial electron transport generates ATP that is essential for the excitability and survival of neurons (Mattson et al., 2008). The expression profile of genes observed in the current study could be attributed to a hyperactive mitochondrial electron transport chain, which could be a compensatory reaction to GWI-induced mitochondrial dysfunction associated with the increased generation of ROS. The elevated expression of mitochondrial-encoded genes in complexes I, II, IV, and V of the respiratory chain in chronic GWI is likely to allow the production of proteins involved in the electron transport chain at greater levels to counter-balance the deviant electron transport chain of mitochondria (Manczak et al., 2005). The changes in complex V genes imply that chronic GWI is associated with an increased energy metabolism. The overexpression of genes mediating ATP synthesis (i.e., *Atp5a1* and *Atp5b*) could also be attributed to the altered energy requirement due to augmented levels of protein degradation (Pecorelli et al., 2013). Increased oxidative phosphorylation in the hippocampus of GWI-rats may also reflect a stress-protection mechanism against the prevailing increased oxidative stress and/or inflammation (Henningsen et al., 2012). A few prior studies have also highlighted the occurrence of mitochondrial dysfunction in GWI, in rodent models as well as in patients. A study by Koslik et al. (2014) demonstrated mitochondrial dysfunction in the calf muscle of patients with GWI. Another study using a mouse model of GWI showed decreased levels of mitochondrial lipids in the brain, implying the reduced activity of electron transport chain (Abdullah et al., 2016), which contradicts the hyperactive mitochondrial respiration suggested by the current findings. The discrepancy between the studies could be attributed to the different time-points of investigation (i.e., at 15–16 months post-exposure in Abdullah et al., 2016 vis-à-vis 6 months post-exposure in the current study, respectively). It is plausible that mitochondrial electron transport chain initially responds to neurotoxic exposure with hyperactivity, following which the generation of increased amounts of ROS, damages the electron transport chain over a period of time, eventually leading to its reduced activity. Additionally, exposure of animals to much higher doses of PB and PM by Abdullah et al. (2016) than doses used in the current study may have played a role.

The augmented expression of genes relevant to oxidative stress and mitochondrial function in GWI-rats occurred in parallel with the enhanced expression of pro-inflammatory genes *Nfkb1, Bcl6, Csf2, IL1a*, and *IL6* and decreased expression of the anti-inflammatory genes *IL4* and *IL10* by 73–88%. Among genes that were up-regulated, the overall increase was >1.5-fold for *Nfkb1, Bcl6, IL6*, and >2.5-fold for *Csf2*. These changes have considerable implications on hippocampal function. *Nfkb1* encodes p50, which is an integral modulator of Nfkb signaling. Nfkb regulates the expression of inflammatory cytokines, chemokines, adhesion molecules, and apoptosis of several cell types (Karin and Lin, 2002; Bourteele et al., 2007). Since deletion of *Nfkb1* can decrease the inflammatory response to brain injury (Rolova et al., 2015) and age-related brain inflammation is linked to augmented Nfkb1 expression (Primiani et al., 2014), it is possible that the enhanced expression of *Nkfb1* plays a role in the persistent inflammation observed in the hippocampus of GWI-rats. It could also be the reason for the enhanced expression of other pro-inflammatory cytokine genes such as Bcl6, Csf2, IL1a, and IL6. The protein coding Bcl6 controls the stability of regulatory T-cells (Sawant et al., 2015). On the other hand, the protein coding *Csf2* (granulocyte-macrophage colony-stimulating factor), is typically upregulated in glioblastoma patients and believed to promote alternative activation of microglia (Sielska et al., 2013). IL1a is a potent inflammatory cytokine and a key mediator of brain inflammation (Brough and Denes, 2015) whereas IL6 is a pro-inflammatory cytokine involved in depression (Kong et al., 2015). Increased depressive-like behavior seen in GWI-rats (Parihar et al., 2013; Hattiangady et al., 2014) may be linked to increased IL6 levels in the hippocampus. Since IL4 and IL10 are anti-inflammatory cytokines, their decreased expression in the hippocampus of GWI-rats is likely to be detrimental for controlling ongoing inflammation (Gadani et al., 2012; Lobo-Silva et al., 2016). IL4 also plays a role in learning and memory. Thus, the pattern of gene expression observed points to a considerable inflammatory activity in the hippocampus at 6 months after exposure to GWIR-Cs and stress. This is consistent with the presence of activated microglia and reactive astrocytes observed in our earlier immunohistochemical study (Parihar et al., 2013). Decreased IL4 concentration has also been reported in the plasma of GWI patients (Khaiboullina et al., 2015).

Moreover, several genes linked to neurodegenerative disorders and/or cognitive and memory function showed an altered expression. These include increased expression of *Mapk1, Mapk3, Ngf, Npac, Narg2*, and *Prkaca* genes, and decreased expression of *Vegfa gene*.

Among genes that were up-regulated, the overall increase was >1.5-fold for *Mapk1, Mapk3, Ngf, and Npac*, and >2.5-folds for *Prkaca*. The proteins encoding these genes have multiple functions. Mapk1 and Mapk3 belong to the MAP kinase family, which are also known as extracellular signal-regulated kinases (ERKs), and are elevated during increased oxidative stress and in neurodegenerative conditions such as Alzheimer’s disease (Gerschütz et al., 2014). Ngf is typically elevated in the brain under inflammatory conditions (Skaper, 2017) whereas Npac regulates p38 MAP kinase activity. On the other hand, increased activation of Prkaca is linked with the growth of cancer, including brain cancer (Yazaki et al., 1996; Koivunen et al., 2006). Vegfa encodes a heparin-binding protein, which is involved in the proliferation of endothelial cells that is essential for angiogenesis. Vegfa also promotes a wide range of neuronal functions, including neurogenesis (Rosenstein et al., 2010). Hence, the decreased expression of Vegfa is detrimental to hippocampal function. Decreased Vegfa concentration has also been observed in the blood of GWI patients (Khaiboullina et al., 2015). Thus, increased levels of Mapk1, Mapk3, Ngf, and Npac along with decreased expression of Vegfa in the hippocampus are consistent with the presence of cognitive, memory and neurogenesis impairments in GWI-rats (Parihar et al., 2013; Hattiangady et al., 2014). Greatly enhanced expression of Prkaca likely suggests an increased vulnerability for developing brain cancer. This is in accordance with the increased incidence of brain cancer observed in GW veterans (White et al., 2016).

Alterations in the expression of genes related to oxidative stress, inflammation, neurodegeneration, cognition, and memory were also associated with increased Nrf2 immunoreactivity in hippocampal neuronal nuclei and activated Nrf2 in the hippocampus in general, in GWI-rats. The transcription factor Nrf2 is a dominant regulator of redox homeostasis that curbs inflammation. Nrf2 generates antioxidant and phase II detoxification enzymes under conditions of augmented oxidative stress, which inherently decreases oxidative stress and buildup of toxic metabolites. Nrf2 regulates the expression of multiple cytoprotective genes that share a *cis*-acting enhancer sequence termed “antioxidant response element,” which include Hmox1 gene coding the antioxidant enzyme heme oxygenase 1, Nqo1 gene coding NADP(H) quinone oxidoreductase, and genes coding enzymes for glutathione metabolism and protein degradation via the proteasome and autophagy routes (Innamorato et al., 2009; Joshi and Johnson, 2012; Lastres-Becker et al., 2012, 2014). Heme oxygenase 1 cleaves heme to release biliverdin-Ixα and carbon monoxide, both of which have anti-inflammatory activity (Cuadrado and Rojo, 2008; Lastres-Becker et al., 2014). Nrf2 activation in the hippocampus in the current study was linked with increased expression of both Hmox1 and Nqo1 genes, implying the activation of endogenous cytoprotective and anti-inflammatory activity. Nrf2 also modulates microglial activation in the brain (Innamorato et al., 2008, 2009; Lastres-Becker et al., 2014). Thus, Nrf2 activation in the chronic phase after exposure to GWIR-Cs could be an innate response to diminish the persistent increased oxidative stress and inflammation in the hippocampus.

Interestingly, changes in the hippocampus of GWI-rats were accompanied with increased oxidative stress and inflammation at the systemic level. The concentration of MDA was enhanced in the serum, 6 months after exposure to GWIR chemicals and stress. Furthermore, elevated levels of multiple cytokines and chemokines, which include Tnfa, IL1b, IL1a, Tgfb, Mip1a, IL6, IP10, Scf, Vegf, Fgfb, IL5, and IL15, were observed in the serum. How do these inflammatory markers relate to biomarkers identified in GWI patients? Systemic inflammation has also been reported in GWI patients (Broderick et al., 2013; Smylie et al., 2013; O’Callaghan et al., 2016). Studies on serum/plasma samples from GWI patients have shown increased levels of IL1b and IL15 on higher fatigue severity days (Parkitny et al., 2015). Furthermore, increased soluble receptor II for Tnf was significantly associated with reduced hippocampal volume in Gulf war veterans (O’Donovan et al., 2015). Moreover, higher numbers of lymphocytes, monocytes, neutrophils and platelets and elevated levels of C-reactive protein, leptin, brain-derived neurotrophic factor and matrix metalloproteinase-9 were seen in GWI patients (Johnson et al., 2016). In addition, analyses of serum from myalgic encephalomyelitis and chronic fatigue syndrome patients (conditions that are considered closer to GWI patients) have revealed decreased levels of IL-16 and IL-7 (Landi et al., 2016). These findings raise an important question as to whether hippocampal dysfunction in GWI is a result of persistent oxidative stress and inflammation at the systemic level, rather than due to focal alterations in the hippocampus itself. Multiple previous studies have shown that systemic inflammation can produce enduring cognitive impairments through immune-to-brain communication (Yamanaka et al., 2017). Examples include cognitive dysfunction in survivors of sepsis (Iwashyna et al., 2010), reduced hippocampal volume and hippocampus-dependent working memory dysfunction due to systemic inflammation sustained during the postnatal period (Malaeb et al., 2014), and microglial activation and impaired long-term potentiation in the hippocampus of mice with peripheral inflammation (Di Filippo et al., 2013). How does systemic inflammation influence brain function? Studies have shown that both hematogenous and neural pathways are conduits of interaction between systemic inflammation and the brain (Dantzer et al., 2008). The hippocampus in particular is more susceptible to systemic inflammation-induced dysfunction, as it displays a higher density of pro-inflammatory cytokine receptors (Jurgens and Johnson, 2012). Furthermore, Ridder et al. (2014) suggested that in conditions such as systemic inflammation, genetic information is transferred from the hematopoietic system to the brain through extracellular vesicles.

## Conclusion and Future Studies

This study validated that unrelenting oxidative stress, mitochondrial impairment, Nrf2 activation, and inflammation can occur in the hippocampus with exposure to relatively low-doses of GWIR-Cs and mild stress in a rat model. Previous studies have demonstrated hippocampus-dependent cognitive, memory and mood impairments in the same animal prototype. Since the various enduring modifications in the hippocampus elucidated in this study can impede cognitive, memory and mood function, potential therapies for GWI would need to alleviate these adverse changes in the hippocampus for improving function. In this context, effective antioxidant and/or anti-inflammatory drug therapies or compounds and small molecules that can directly or indirectly modulate oxidative stress and inflammation signaling pathways or repair mitochondrial dysfunction in the hippocampus have promise to improve the quality of life in GWI patients. Nonetheless, this study also revealed that persistently increased oxidative stress and inflammation are not restricted to the hippocampus but global in nature. This was evidenced by considerable systemic inflammation typified by enhanced levels of multiple pro-inflammatory cytokines in the serum, consistent with results of multiple blood biomarker studies performed in GWI patients. This finding raises the possibility that hippocampal dysfunction in GWI is one of the adverse outcomes of persistently elevated oxidative stress and inflammation at the systemic level. It is plausible that incessantly higher levels of pro-inflammatory cytokines and chemokines in the circulating blood cause brain dysfunction. Therefore, a larger question to address in the future is whether long-term suppression/modulation of systemic inflammation would be efficacious for improving brain function in GWI patients, in addition to relieving other symptoms such as sickness-like behavior and chronic pain.

## Author Contributions

AS, GS, and BH designed research; GS, BH, DU, AB, SA, BS, and MK performed research and collected data. AS, GS, BH, DU, and AB analyzed and interpreted data; GS and AS wrote the paper. All authors gave inputs to the manuscript text and approved the final version of the manuscript.

## Disclaimer

The contents of this article suggest the views of authors and do not represent the views of the United States Department of Veterans Affairs, Department of Defense or the United States Government.

## Conflict of Interest Statement

The authors declare that the research was conducted in the absence of any commercial or financial relationships that could be construed as a potential conflict of interest.
